# Evidence for coseismic subsidence events in a southern California coastal saltmarsh

**DOI:** 10.1038/srep44615

**Published:** 2017-03-20

**Authors:** Robert Leeper, Brady Rhodes, Matthew Kirby, Katherine Scharer, Joseph Carlin, Eileen Hemphill-Haley, Simona Avnaim-Katav, Glen MacDonald, Scott Starratt, Angela Aranda

**Affiliations:** 1 California State University, Fullerton, Geological Sciences, Fullerton, CA 92831, USA; 2 U.S. Geological Survey, Pasadena, CA 91106, USA; 3 Hemphill-Haley Consulting, McKinleyville, CA 95519, USA; 4 University of California, Los Angeles, Geography, Los Angeles, CA 90095, USA; 5 U.S. Geological Survey, Menlo Park, CA 94025, USA

## Abstract

Paleoenvironmental records from a southern California coastal saltmarsh reveal evidence for repeated late Holocene coseismic subsidence events. Field analysis of sediment gouge cores established discrete lithostratigraphic units extend across the wetland. Detailed sediment analyses reveal abrupt changes in lithology, percent total organic matter, grain size, and magnetic susceptibility. Microfossil analyses indicate that predominantly freshwater deposits bury relic intertidal deposits at three distinct depths. Radiocarbon dating indicates that the three burial events occurred in the last 2000 calendar years. Two of the three events are contemporaneous with large-magnitude paleoearthquakes along the Newport-Inglewood/Rose Canyon fault system. From these data, we infer that during large magnitude earthquakes a step-over along the fault zone results in the vertical displacement of an approximately 5-km^2^ area that is consistent with the footprint of an estuary identified in pre-development maps. These findings provide insight on the evolution of the saltmarsh, coseismic deformation and earthquake recurrence in a wide area of southern California, and sensitive habitat already threatened by eustatic sea level rise.

Coastal southern California is prone to earthquake hazards and is under additional threat from eustatic sea level rise coupled with gradual tectonically driven subsidence^1^,^2^,^3^. Abrupt coseismic subsidence in coastal areas can compound these risks by producing instant vertical displacement affecting geomorphic processes over wide areas^4^,^5^,^6^,^7^. Submergence of coastal wetlands due to coseismic subsidence results in sharp changes in lithology and paleoecology that commonly reflect a deepening of the depositional environment^4^,^5^,^6^,^8^,^9^. Here, we evaluate the Seal Beach saltmarsh in southern California that contains multiple buried peaty and organic-rich mud layers, which is different when compared to other southern California coastal wetlands^3^,^10^,^11^. We hypothesize that peaty and organic-rich intertidal deposits, buried by coarse-grained sediments containing mostly sparse allochthonous freshwater diatoms, demonstrates that the saltmarsh coseismically subsided three times during the late Holocene. The distribution of the buried intertidal layers suggests that the saltmarsh occupies a fault-bound basin along the Newport-Inglewood fault and that during large magnitude earthquakes an approximately 5-km^2^ area between Landing Hill and Bolsa Mesa (Figs 1 and 2) abruptly subsides.

The Newport-Inglewood fault is a seismically active structurally complex right-lateral strike-slip fault comprised of en echelon fault strands, oil-bearing anticlines, and subsidiary fault segments exhibiting normal and reverse displacement^12^,^13^,^14^,^15^. The fault is located along the western margin of the Los Angeles Basin, extending onshore for ~75 km from the City of Beverly Hills in the north to the City of Newport Beach in the south. From there the fault continues offshore and connects to the Rose Canyon fault^16^, which roughly parallels the coastline for ~90 km into the San Diego region (see Supplementary Fig. S1). Recent characterization of step-overs along the fault system offshore indicates earthquakes could rupture in single events between the regions of San Diego and Newport Beach, yielding large magnitude (M7.5) earthquakes^17^. The Newport-Inglewood/Rose Canyon fault system thus transects some of the most developed areas of coastal southern California.

The Seal Beach saltmarsh is the last undeveloped portion of the Anaheim Bay estuary that once covered a large area in Sunset Gap near Seal Beach, California (Figs 1 and 2). Prior to development, the saltmarsh had large fringing freshwater wetlands, salt flats, and alkali meadows^18^,^19^ (Fig. 2). Reclaimed areas of the estuary proximal to the fault are now occupied by military, municipal and industrial infrastructure including the U.S. Naval Weapons Station Seal Beach, Huntington Harbor, the 965 acre Seal Beach National Wildlife Refuge, and an active oil-extraction operation (Figs 1 and 2). Paleoseismology and paleoenvironmental analyses of the saltmarsh offer an opportunity to better understand late Holocene earthquake occurrence on an important and poorly understood southern California fault system^20^,^21^.

## Results

We collected 55 sediment cores in the saltmarsh to explore the geologic history of the wetland (Fig. 2, see Supplementary Table S2). Most sediment cores collected were reconnaissance gouge cores, examined visually to look for sharp lithological changes and to identify locations in the saltmarsh to collect large diameter cores for sedimentological analyses (see Supplementary Fig. S3). Lithological descriptions of the reconnaissance gouge cores combined with sedimentological analyses of four vibracores and one piston core indicate that four distinct lithostratigraphic units extend across the saltmarsh (Figs 3 and 4). We describe the lithostratigraphic units based on the sedimentological analyses conducted on the large diameter cores. The basal unit consists of gray organic-poor silty sand containing sparse mica and scattered and clustered shell fragments, which is overlain by interlayered peaty to organic-rich mud or coarse-grained micaceous silty sand. Marine diatoms occur in the basal unit, and most are broken and show abrasion at the edges (Fig. 5, see Supplementary Data S4). We also found fragmented and abraded disarticulated pieces of a bivalve, *Chione californiensis*, which lives in mud flats, estuarine environments and shallow marine settings^22^. The fragmented and abraded diatoms and shells suggest that the marine organisms were living in an environment exposed to wave energy, indicating the basal unit may represent a period when the site was a shallow marine embayment.

To understand the ecological changes associated with the layers above the basal unit, we paired microfossil analyses with sedimentological data in two representative vibracores, 02VC and 18VC (Figs 5 and 6, see Supplementary Data S4 and S5), which are separated in the saltmarsh by approximately 650 m (Fig. 2). As illustrated in Fig. 5, the transition in 02VC across the sharp depositional contact at 222.5 cm depth is characterized by a change from the expected saltmarsh deposits to organic-poor micaceous silty sand, accompanied by key changes in the microfossils. For example, at 229 cm we document intertidal saltmarsh deposits characterized by preservation of abundant intertidal diatoms and foraminifera in organic-rich silt with low magnetic susceptibility, similar to the deposits on the saltmarsh surface today (Fig. 5, see Supplementary Data S4). At 227 cm, the deposits show a slight drop in the abundance of intertidal diatoms and a decrease in the intertidal foraminifera abundance by ~45%, commensurate with the appearance of fresh-to-fresh brackish (f-fb) diatoms. The f-fb diatoms are an important indicator, as they are abraded and fractured and likely allochthonous, suggesting introduction of a freshwater source to the saltmarsh. The f-fb diatom taxa preserved are consistent with subaerial and shallow freshwater and alkali environments; possibly similar to those historically mapped fringing the Anaheim Bay estuary (Fig. 2). The sediments in this interval show an increase in silt and magnetic susceptibility and decrease in organic content, changes also consistent with a shift in paleoenvironmental conditions that occurs between 229 cm and 227 cm.

Indications of freshwater and tidal water mixing persist up the core (Fig. 5). At 225 cm, abundant allochthonous f-fb diatoms occur with sparse intertidal diatoms, and no foraminifera are present. At 222.5 cm there is a sharp depositional contact where the underlying organic-rich silt is buried by a thick (~100 cm) layer of organic-poor coarse-grained micaceous silty sand. By 200 cm the limited intertidal diatoms are absent; the only remaining microfossils are sparse abraded and fractured f-fb diatoms (Fig. 5). At ~100 cm depth, the sediment starts to return to saltmarsh deposits.

Microfossil analyses in 02VC across the lithologic transition at 40 cm indicate that muddy peat containing intertidal diatoms is overlain by organic-rich silt containing f-fb diatoms and intertidal diatoms (see Supplementary Data S5). At 324 cm, abundant intertidal diatoms occur in organic-rich sandy silt (Fig. 5). An abrupt lithological transition occurs at 320 cm. A spike in MS and decreased organic content and percent sand characterizes the transition. At 305 cm, the lithostratigraphic unit contains abundant intertidal diatoms and sparse allochthonous f-fb diatoms hinting at the introduction of freshwater at the site. By 295 cm, f-fb diatoms are more abundant than intertidal diatoms. At approximately 280 cm, coarse-grained micaceous sandy silt buries the organic-rich mud. The character of this transition is similar to what occurs shallower in the core across the sharp depositional contact at 222.5 cm (Fig. 5).

Microfossil analyses in 18VC indicate that coarse-grained micaceous silty sand deposits completely devoid of intertidal diatoms and foraminifera abruptly bury relic intertidal deposits at three distinct depths: 45 cm, 198 cm, and 253.5 cm (Fig. 6). Based on the combined analyses in 02VC and 18VC, we identify three separate events (E1, E2, and E3) in the vibracores where similar lithologic transitions occur and paleoecological data indicate a change in depositional environment. Furthermore, using stratigraphic position and sedimentological observations documented in the reconnaissance cores, we correlate the events across the saltmarsh (Figs 3 and 4). Radiocarbon dating of charcoal, organic remains and shells indicates the events occurred 2085-1910 cal yrs BP (E3), 1603-1302 cal yrs BP (E2), and 632-363 cal yrs BP (E1) (Fig. 7, see Supplementary Table S6).

## Discussion

We interpret the lithostratigraphic sequence in the Seal Beach saltmarsh as recording three abrupt paleoenvironmental changes from saltmarsh deposits, to predominantly freshwater influenced silty sand, and then a return to a saltmarsh environment. What historic and geologic factors could contribute to the deposition and preservation of these events? During the winter of 1938, a historically large flood blanketed the coastal plain of Los Angeles and Orange County with >1-m thick deposits of silty sand^12^, suggesting the possibility that the thick micaceous silty sand deposits in the saltmarsh could be the remains of terrestrial flooding events. However, radiocarbon ages establish that the uppermost silty sand deposits at Seal Beach predate historic flooding events and suggest the estuary may not be a good recorder of major floods^12^,^23^. Thus, preservation of the silty sand units likely requires production of accommodation space or other mechanisms to isolate the silty sand from coastal erosion and sea level rise. Vibracore 02VC shows that intertidal marsh deposits originally deposited at sea level are now at a depth of ~350 cm below the modern marsh surface (Fig. 5). These marsh deposits have a maximum age of about 2700 cal BP (Fig. 7), leading to an average accumulation rate in 02VC of about 1.3 mm/yr. This rate exceeds the late Holocene relative sea level rise rate of 0.8 +/− 0.3 mm/yr recently established for southern California^24^ indicating subsidence could be controlling the preservation of the silty sand units. Additionally, the sedimentary sequence at Seal Beach is different than the wetland sites used for the sea level curve, which show relatively constant marsh aggradation as sea level rises^24^. This suggests that a feature local to the Seal Beach saltmarsh is needed to produce and preserve the abrupt transitions indicated by events E1, E2, and E3.

**Figure 1:**
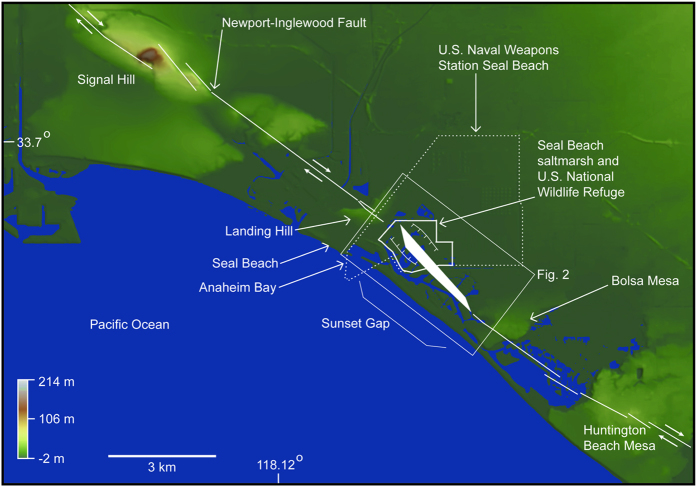
National Elevation Dataset (10 m) base map showing structural complexity and geographic points of interest along the Newport-Inglewood fault. Small white arrows along the fault show sense of movement. Fault traces are from State of California Special Studies Zones Seal Beach Quadrangle, 1986^60^. White polygon illustrates right step in trace of fault and white lines with inward hatches indicate zone and pattern of subsidence. Topographic data was obtained from the U.S. Geological Survey National Elevation Dataset, 1/3 arc second data via The National Map Viewer^61^, modeled using QT Modeler v.8.0 and modified in Adobe Illustrator CC v.20.1.0.

**Figure 2:**
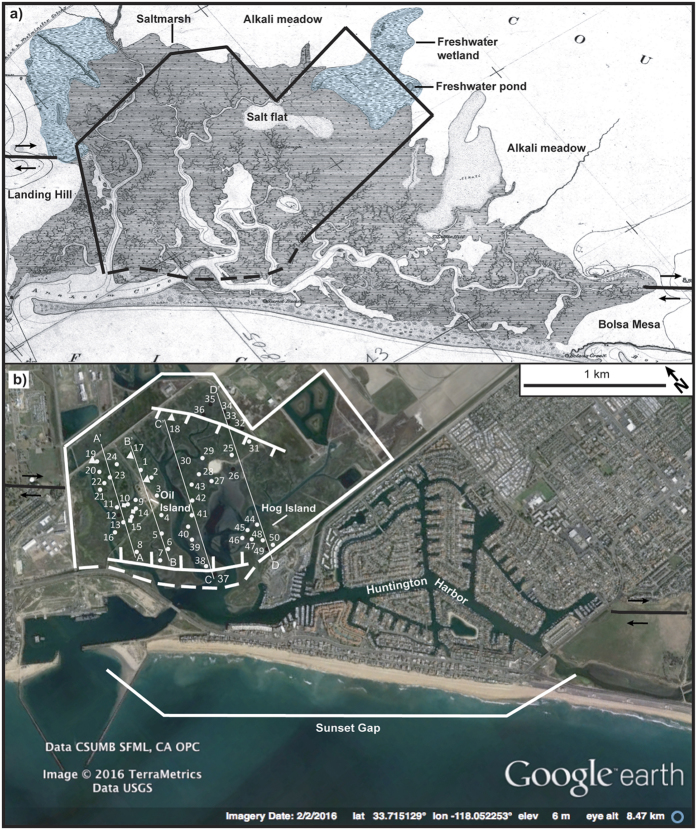
Images show predevelopment (1873 AD) and developed (2016 AD) portions of the Anaheim Bay estuary. (**a**) T-sheet 1345^18^ showing the Anaheim Bay estuary depositional environments. Black lines with arrows are fault surface traces showing sense of fault movement. Base image (T-sheet 1345^18^) modified from http://nosimagery.noaa.gov/images/shoreline_surveys/survey_scans/T-1345.JPG^63^ in Adobe Illustrator CC v.20.1.0. (**b**) Numbers and black and white solid circles indicate sediment core sites. Circles show reconnaissance gouge cores, squares are piston cores, and triangles are vibracores. White circles show cores where buried peaty to organic-rich sediment was observed. Black circles indicate cores without buried peaty to organic-rich sediment. Solid thin white lines indicate arbitrary core northeast trending transect lines shown in Supplementary Fig. S2. Bold white lines with inward hatches indicate zone and pattern of subsidence. Base imagery from Google Earth Pro v.7.1.2.2041, Data CSUMB, SFML, CA OPC, USGS, Image: © 2016 TerraMetrics and modified in Adobe Illustrator CC v.20.1.0.

**Figure 3:**
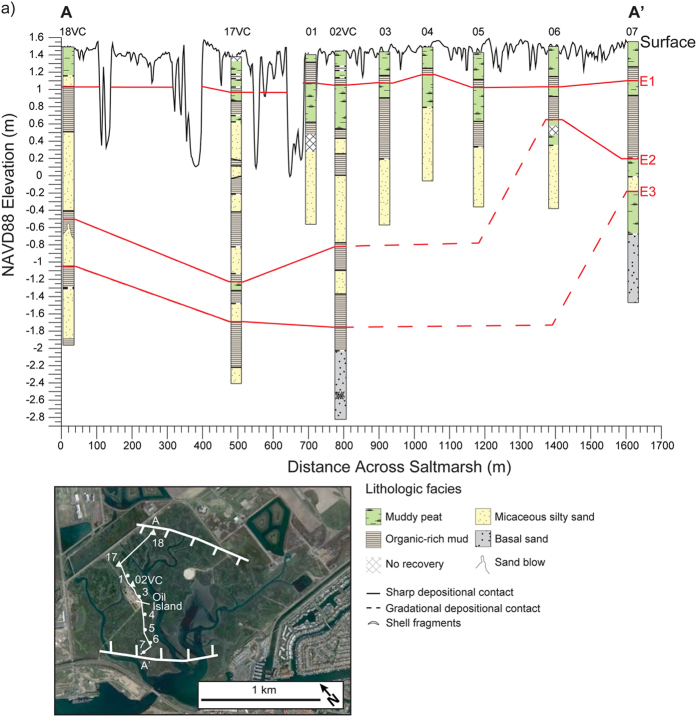
Image shows correlated saltmarsh lithostratigraphy. Seal Beach saltmarsh lithostratigraphy across transect line A-A’ shown in base map (base map taken from Fig. 2). Red lines are correlated burial events E1–E3. Base imagery map from Google Earth Pro v.7.1.2.2041, Data CSUMB, SFML, CA OPC, USGS, Image: © 2016 TerraMetrics and modified in Adobe Illustrator CC v.20.1.0.

**Figure 4:**
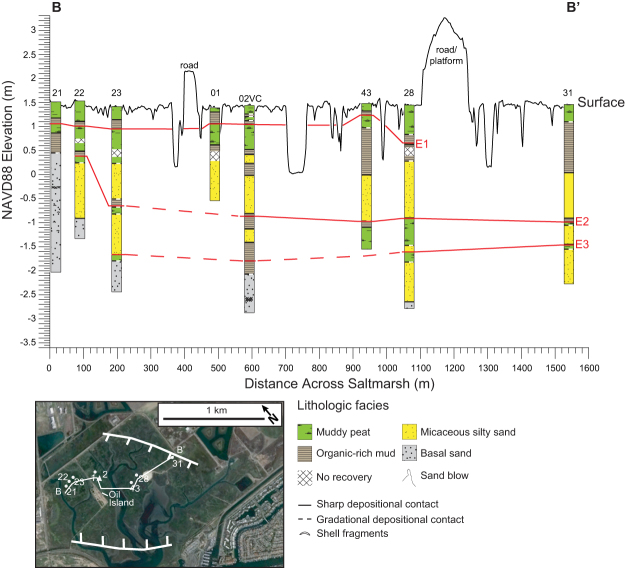
Image shows correlated saltmarsh lithostratigraphy. Seal Beach saltmarsh lithostratigraphy across transect line B-B’ shown in base map (base map taken from Fig. 2). Red lines are correlated burial events E1–E3. Base imagery map from Google Earth Pro v.7.1.2.2041, Data CSUMB, SFML, CA OPC, USGS, Image: © 2016 TerraMetrics and modified in Adobe Illustrator CC v.20.1.0.

The geometry of the Newport-Inglewood fault within Sunset Gap (Fig. 1) provides a mechanism that could produce coseismic subsidence, episodically increasing accommodation space and enabling preservation of the micaceous coarse-grained sediment. Extensive oil and water well data in the region show that within Sunset Gap primary aquifers 100–200 m deep are folded into a broad structural low on the northeast side of the fault between Landing Hill and Bolsa Mesa^25^. Upper Miocene and early Pliocene units also step down on the northeast side of the fault zone at the saltmarsh^15^, providing evidence that at a broad scale, both older and more recent deposits show persistent vertical motion isolating the landward side of the fault as a consequence of the tectonic setting. At an even more local scale, the geometry of the fault zone between Bolsa Mesa and Landing Hill would also tend to accentuate subsidence in the center of the saltmarsh due to a small (5°) right step in the fault (Figs 1 and 2). The step in the fault zone could be resolved as a small pull-apart basin at least 1 km wide, similar in scale to other steps and bends in the fault such as a graben northwest of Huntington Beach Mesa where paleoearthquakes are documented^26^ and a small push-up ridge at Signal Hill located to the NW (Fig. 1).

The work of^4^ was modified by^5^,^6^ in order to establish criteria indicative of coseismic subsidence in wetland environments straddling extensional step-overs along strike slip faults. The criteria established are: (i) an abrupt upper sedimentary contact; (ii) diatom evidence for relative sea level change; (iii) evidence for sustained submergence and/or rapid aggradation; (iv) graben or releasing fault geometry; (v) wide lateral extent of submergence; and (vi) synchrony with other paleoseismic events. Comparing the Seal Beach saltmarsh data set to these criteria suggests that the abrupt burial events are consistent with the hypothesis that earthquakes along the Newport-Inglewood fault system resulted in coseismic subsidence of the relic wetlands surface three times in the past 2000 years, occurring approximately every 700 years (Fig. 7).

**Figure 5:**
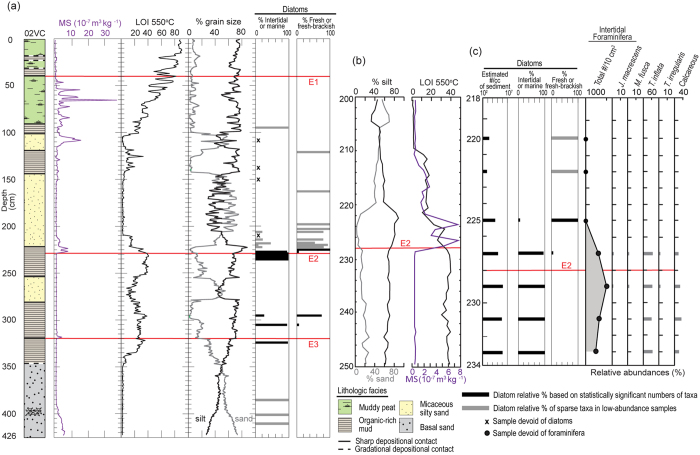
Graphs show sedimentological and paleoecological data for 02VC. (**a**) Results from left to right: core log; magnetic susceptibility (MS); loss-on ignition (LOI), percent grain size; and diatom relative abundances (%). Red lines show the burial event horizons E1–E3. (**b**) Expanded view of results for LOI and MS between 200–250 cm. (**c**) Variability in diatom and foraminiferal distributions between 218–234 cm, spanning event horizon E2. The graphs were created in Adobe Illustrator CC v.20.1.0.

**Figure 6:**
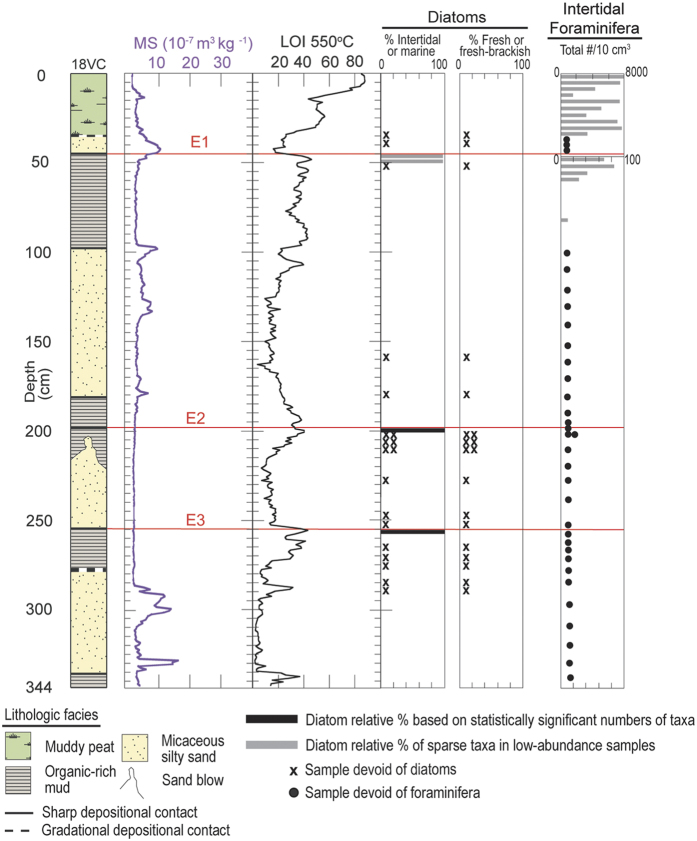
Graphs show sedimentological and paleoecological data for 18VC. Results from left to right: core log; graphs of magnetic susceptibility (MS); loss-on ignition (LOI), and diatom and foraminifera data. Note in the foraminifera graph, the change in scale at 50 cm. At the microfossil sample sites below 200 cm, the two symbols represent results of analyses from both the organic-rich mud and micaceous silty sand. Red lines show the event horizons E1–E3. The graphs were created in Adobe Illustrator CC v.20.1.0.

**Figure 7:**
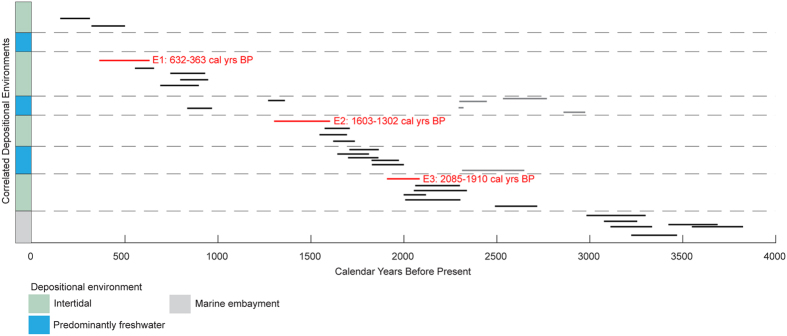
Radiocarbon ages for correlated depositional environments in the Seal Beach saltmarsh. Black bars are the 2-sigma radiocarbon ages in Calendar Years Before Present. Red bars are the 2-sigma radiocarbon ages for events E1–E3. Gray bars are the 2-sigma radiocarbon ages that are significantly older than samples from layers below likely reflecting long transport times before deposition as detritus in the saltmarsh. The age model was produced in OxCal v4.2^58^ and modified in Adobe Illustrator CC v.20.1.0.

**Figure 8:**
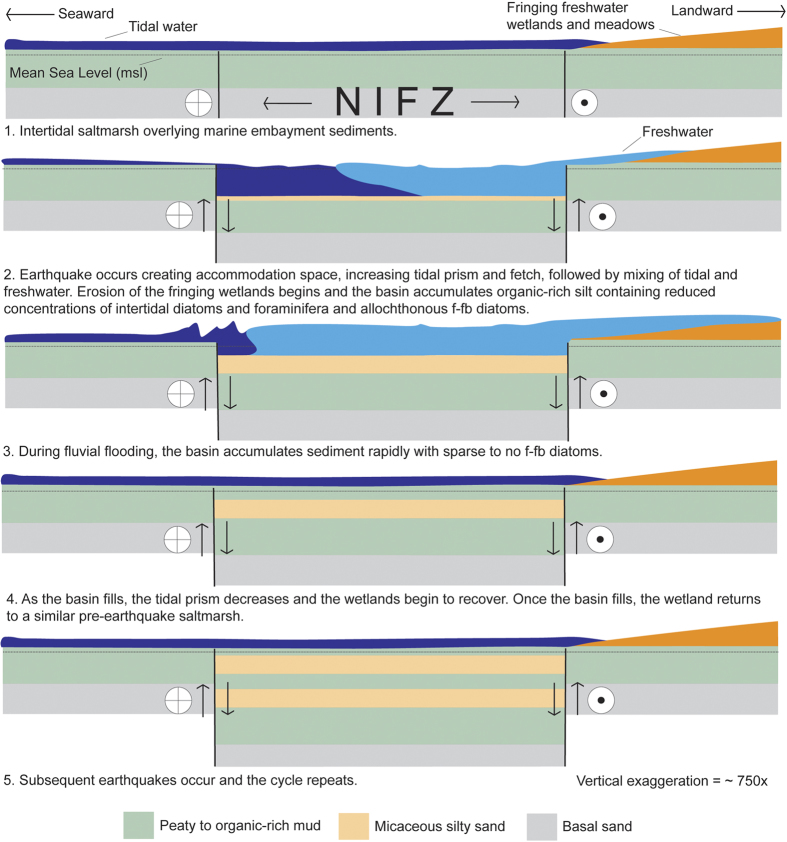
Simplified schematic diagram showing how earthquakes created accommodation space and controlled the evolution of the saltmarsh during the late Holocene.

The earthquakes episodically lower the saltmarsh where a small 1 km wide right step occurs in the fault trace, instantly creating accommodation space and increasing the tidal prism and fetch (Fig. 8). Immediately following the subsidence, mixing of tidal water and freshwater begins reducing the concentrations of intertidal diatoms and foraminifera that accumulate in the submerged basin with sparse allochthonous f-fb diatoms. As the surrounding freshwater wetlands erode, intertidal microfossils disappear and only f-fb diatoms are preserved in low concentration. The basin is subsequently filled during freshwater floods by sediment that is nearly devoid of microfossils or does not contain microfossils (Figs 5 and 6). Once the accommodation space created by the coseismic subsidence fills, the wetland returns to a similar pre-earthquake saltmarsh (Fig. 8).

Comparison of the proposed paleoearthquake record at Seal Beach to regionally documented earthquakes reveals interesting patterns in the size and frequency of potential earthquakes on the Newport-Inglewood fault. The largest historic earthquake on the fault is the 1933 (M_L_ = 6.3) Long Beach earthquake, which ruptured a 13–16 km-long section of the fault between the cities of Newport Beach and Long Beach^27^,^28^. The earthquake resulted in extensive structural damage in the Los Angeles region and 120 deaths^27^,^28^. There was no surface rupture mapped during field investigations following the 1933 earthquake, although liquefaction and lateral spreading did occur^13^,^20^,^27^. Cracking was reported “along and near the structural [fault] zone” in the saltmarsh^15^ but we observe no evidence of the 1933 earthquake in the cores, consistent with minimal land-level changes and no lateral offsets measured during this moderate sized earthquake. Consequently, the M_L_ 6.3 historic earthquake provides a minimum estimate for the size of the paleoearthquakes, since the historic earthquake was not associated with any subsidence.

The most recent earthquake (E1) identified in the saltmarsh is contemporaneous with a surface-rupturing earthquake documented along the Rose Canyon fault, during which approximately 3 m of slip occurred^29^,^30^. If the 3 m offset is the average for this Rose Canyon event, empirical scaling^31^ permits that the rupture could be ~225-km long, extending northwest of the saltmarsh, providing both temporal and geomorphic evidence for major (Mw7.6) earthquakes on the system. Alternatively, considering dating limitations at each site and Coulomb modeling^17^, E1 could represent the northernmost rupture of a sequence of intermediate sized earthquakes that sequentially ruptured up the coast^21^. The only long-term estimate of paleoearthquake recurrence for the Newport-Inglewood fault is from near the Huntington Beach oil field, where^26^ used continuous cone penetrometer borings to locate offsets in stratigraphic units in a small graben on the fault. They interpret five Holocene earthquakes. Of this record, only the youngest earthquake near the Huntington Beach oil field could be correlative with one of the events in the Seal Beach saltmarsh.

We propose that the saltmarsh coseismically subsided three times in the past 2000 years. It is possible that the entire historic Anaheim Bay estuary is related to coseismic deformation along the Newport-Inglewood fault (Figs 1 and 2). Future earthquakes that result in subsidence of the saltmarsh may present serious hazards to the U.S. Naval Weapons Station Seal Beach, Seal Beach National Wildlife Refuge, Huntington Harbor, and other southern California coastal communities.

## Methods

### Field Techniques

Reconnaissance cores were described visually, specifying sediment type, color, presence/no presence of mica flakes, and the relative amount of organic material. For deeper probing at a site, 1-m long extension rods were attached to the end of the core barrel and the procedure was repeated until penetration was no longer possible. In order to collect sediment for laboratory analysis, we collected three Livingstone piston cores (5.08 cm diameter) and four vibracores (7.62 cm diameter) (Fig. 2).

### Quantitative Sediment Analyses

The large diameter cores were cut in half lengthwise, scraped clean with a razorblade (perpendicular to the length of the core), measured, photographed, visually described, and subsampled at 1-cm contiguous intervals into 1-cm^3^ plastic cubes. Changes in lithology were determined through measurement of magnetic susceptibility^32^, loss-on-ignition^33^ (% total organic matter), and grain size. Mass magnetic susceptibility was determined using a Barrington MS2 Magnetic Susceptibility meter at 0.465 kHz in SI units of 10^−7^ m^3^ kg^−1^. Grain size analysis was performed at 1-cm sample resolution using a Malvern Mastersizer 2000 laser diffraction grain size analyzer. Prior to running the grain size analysis, the samples were treated with 25–50 mL of 30% H_2_O_2_ to obliterate organic matter, 10 mL 1 N HCl to dissolve carbonates, and 10 mL 1 N NaOH to eliminate biogenic silica. The grain size analyzer’s performance was measured regularly every 10th sample using a 1–16 micron standard tuff.

### Diatoms

To remove organic material from samples, approximately 1–3 cm^3^ of sediment was placed in a beaker with enough 30% H_2_O_2_ added to submerge the sample. After the reaction completed, the sample was rinsed clean by filling the beaker with distilled H_2_O and left to settle for at least 1 hr before decanting. This step was repeated several times to remove all remaining H_2_O_2_ as well as clays in suspension. The material was then transferred to a graduated tube and diluted 10:1 with distilled water. An aliquot of the cleaned sample was withdrawn using a mechanical pipette, and transferred to a glass coverslip and allowed to dry. The cover slip is permanently mounted to a glass slide with a mounting medium applicable for diatom studies; Naphrax mounting medium was used in this project.

Evaluation of modern samples from the vegetated saltmarsh surface and tidal mud flat indicated that prominent taxa are similarly distributed in both environments (see Supplementary Data S4). A few diatom taxa show a stronger affinity for either the mud flat (e.g., *Achnanthes intermedia*) or saltmarsh (e.g., *Nitschia fasciculata*), but many clearly occur in both environments. Thus, for the purposes of this study, no effort was made to differentiate between deposits that may have accumulated on a tidal mud flat from those originating in a vegetated saltmarsh environment.

Diatoms were abundant in the modern intertidal samples, but abundance varied widely in down core samples. For all samples, an attempt was made to count at least several hundred valves to provide reliable comparisons between different stratigraphic units. This was not possible for some down core deposits that contained only sparse, presumably reworked valves, or that were devoid of diatoms. Results are expressed as concentration in estimated valves per cc of sediment, as well as relative percentage of the total number counted per sample. Diatoms were identified to species level whenever possible using^34^,^35^,^36^,^37^,^38^,^39^,^40^,^41^,^42^,^43^,^44^,^45^,^46^,^47^,^48^.

### Foraminifera

To remove plant macrofossils and fine-grained material, samples consisting of 1.2–4.1 cm^3^ of sediment were wet-sieved through 500 μm and 63 μm sieves. The >500 μm fraction was examined for larger foraminifera before being discarded. To split the foraminiferal, the fraction between 63 and 500 μm was subdivided into eight equal aliquots using wet splitter^49^,^50^. Where possible, at least 250 specimens were wet-counted under a stereoscopic binocular microscope using reflected light. Taxonomic identifications follow^50^,^51^,^52^,^53^,^54^,^55^. Juvenile specimens of *Trochammina inflata, Jadammina macrescens* that were difficult to distinguish from each other because of their small sizes were lumped into a single group and assigned as juvenile Trochamminids. Specimens of the genus *Ammobaculites* were combined into a single group, because these species were often broken making it difficult to identify them to the species level^54^,^55^. All counts were expressed as numerical abundance of living and dead foraminiferal numbers per 10-cm^3^ of bulk sediment and as a relative abundance (%) of species out of the total group.

### Radiocarbon analysis

Accelerator Mass Spectrometry radiocarbon dating was performed on 35 samples selected from cores 02VC, 18VC, 19VC, and 14PC (see Supplementary Table S6). Shell samples were picked directly from the sediment cores with metal tweezers, rinsed with deionized water and placed in labeled glass vials to dry before being sent for analysis. To sample for charcoal and organic material, approximately 1-cm^3^ of sediment was removed from the cores using a metal spatula while attempting to avoid large vertical roots that could be visually traced extending downward in the core from the surface of the saltmarsh. The sediment was placed in a 125 m sieve, rinsed with deionized water, and charcoal and organic material for dating was picked using a binocular microscope. The samples were placed in labeled glass vials and dried before being sent for analysis.

The radiocarbon samples were analyzed at either the Keck Carbon Cycle AMS Facility at the University of California, Irvine, or at the Center for Acceleration Mass Spectrometry at the Lawrence Livermore National Laboratory (see Supplementary Table S6). The radiocarbon ages obtained on samples of vegetated plant material reflect the initiation of photosynthesis in the saltmarsh or at a time out of the saltmarsh prior to deposition as detritus. Modern roots injected below the saltmarsh surface may have contaminated detrital material or material growing *in-situ*. Thus, we infer that stratigraphically “young” ages result from samples contaminated by roots introducing younger carbon to the sample. We infer that “old” stratigraphic units are detrital, resulting from residence time within the environment prior to deposition in the saltmarsh^56^. Shells were corrected for the marine reservoir effect using the local reservoir correction for Anaheim Slough^57^. In order to develop a chronological sequence of the abrupt changes in the depositional environments of the saltmarsh, and constrain the timing of the earthquakes, we combined twenty-seven laboratory reported radiocarbon ages obtained from stratigraphically correlated deposits using the radiocarbon calibration program, OxCal v4.2^58^ IntCal13 and Marine13^59^ (see Supplementary Data S7). OxCal v4.2^58^ applies Bayesian statistics that integrate chronological restrictions to better constrain the calibrated radiocarbon probability distributions. Reported radiocarbon ages of the charcoal, plant material, and shells are reported in calibrated years BP^59^.

## Additional Information

**How to cite this article:** Leeper, R. *et al*. Evidence for coseismic subsidence events in a southern California coastal saltmarsh. *Sci. Rep.* **7**, 44615; doi: 10.1038/srep44615 (2017).

**Publisher's note:** Springer Nature remains neutral with regard to jurisdictional claims in published maps and institutional affiliations.

## Supplementary Material

Supplementary Information

Supplementary Data S4
