# Supplementary material for: Evidence for coseismic subsidence events in a southern California coastal saltmarsh
Source: Sci Rep. 2017 Mar 20;7:44615. doi: 10.1038/srep44615 (PMC5357896; doi:10.1038/srep44615)
Supplement: Supplementary Information [file srep44615-s1.pdf]

Evidence for coseismic subsidence events in a southern California coastal saltmarsh

Robert Leeper[1, 2, *], Brady Rhodes[1], Matthew Kirby[1], Katherine Scharer[2], Joseph Carlin[1], Eileen Hemphill-Haley[3], Simona Avnaim-Katav[4], Glen MacDonald[4], Scott Starratt[5], and Angela Aranda[1]

Supplementary Information

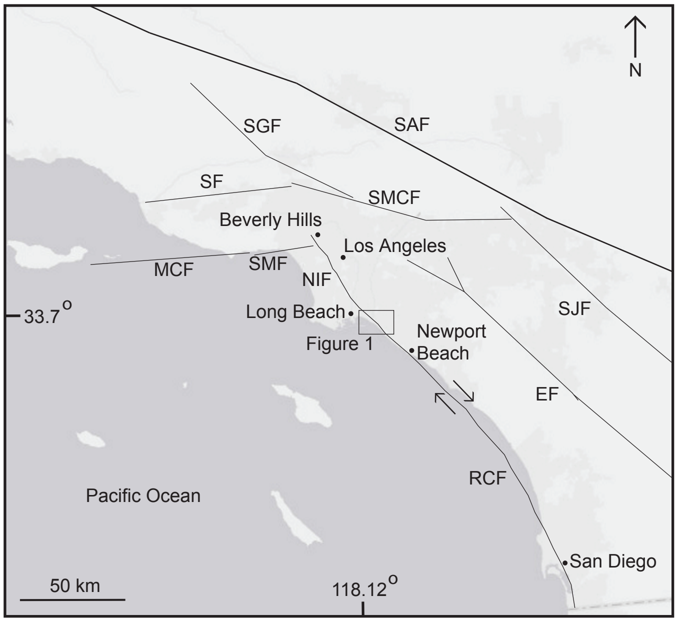

Supplementary Figure S1. Simplified regional fault map of southern California. Faults shown include the San Andreas (SAF), San Gabriel (SGF), Simi (SF), Santa Monica (SMF), Malibu Coast (MCF), Sierra Madre-Cucamonga (SMCF), San Jacinto (SJF), Elsinore (EF), Newport-Inglewood (NIF), and Rose Canyon (RCF). Base imagery from (http://earthquake.usgs.gov/earthquakes/map/) and modified in Adobe Illustrator CC v.20.1.0.

Supplementary Table S2. Locations of reconnaissance cores, piston cores (PC), and vibracores (VC)

Core I.D.	Zone	Easting	Northing	Latitude	Longitude	Core length (cm)
1	11S	0400004	3734019	33°44'29.40"N	118° 4'46.37"W	191
2/02VC	11S	0400025	3733917	33°44'26.13"N	118° 4'45.51"W	287/426
3	11S	0400018	3733795	33°44'22.14"N	118° 4'45.73"W	200
4	11S	0399969	3733677	33°44'18.29"N	118° 4'47.55"W	153
5	11S	0399880	3733563	33°44'14.56"N	118° 4'51.00"W	177
6	11S	0399787	3733375	33°44'8.46"N	118° 4'54.50"W	184
7	11S	0399611	3733301	33°44'5.96"N	118° 5'1.31"W	300
8	11S	0399528	3733559	33°44'14.34"N	118° 5'4.64"W	200
9	11S	0399830	3733891	33°44'25.22"N	118° 4'53.04"W	270
10/PC10	11S	0399716	3733870	33°44'24.50"N	118° 4'57.46"W	244/238
11	11S	0399649	3733904	33°44'25.58"N	118° 5'0.08"W	158
12	11S	0399581	3733853	33°44'23.90"N	118° 5'2.70"W	151
13	11S	0399612	3733777	33°44'21.45"N	118° 5'1.46"W	159
14/PC14	11S	0399786	3733793	33°44'22.02"N	118° 4'54.71"W	170/384
15/PC15	11S	0399643	3733748	33°44'20.52"N	118° 5'0.25"W	264/235
16	11S	0399508	3733750	33°44'20.53"N	118° 5'5.49"W	171
17VC	11S	0400038	3734210	33°44'35.65"N	118° 4'45.08"W	377
18VC	11S	0400548	3734311	33°44'39.10"N	118° 4'25.31"W	344
19/19VC	11S	0399700	3734365	33°44'40.56"N	118° 4'58.28"W	372/400
20	11S	0399704	3734204	33°44'37.94"N	118° 4'58.09"W	378
21	11S	0399591	3734114	33°44'32.38"N	118° 5'2.42"W	351
22	11S	0399668	3734140	33°44'33.25"N	118° 4'59.43"W	283
23	11S	0399768	3734195	33°44'35.07"N	118° 4'55.57"W	381
24	11S	0399848	3734258	33°44'37.14"N	118° 4'52.49"W	500
25	11S	0400738	3733677	33°44'18.58"N	118° 4'17.67"W	300
26	11S	0400667	3733565	33°44'14.92"N	118° 4'20.38"W	184
27	11S	0400474	3733598	33°44'15.93"N	118° 4'27.89"W	400
28	11S	0400422	3733696	33°44'19.09"N	118° 4'29.95"W	418
29	11S	0400538	3733823	33°44'23.25"N	118° 4'25.50"W	247
30	11S	0400393	3733813	33°44'22.85"N	118° 4'34.63"W	215
31	11S	0400898	3733746	33°44'20.88"N	118° 4'11.48"W	369
32	11S	0400879	3733830	33°44'23.60"N	118° 4'12.25"W	300
33	11S	0400878	3733946	33°44'27.36"N	118° 4'12.34"W	187
34	11S	0400907	3734033	33°44'30.20"N	118° 4'11.24"W	300
35	11S	0400870	3734140	33°44'33.66"N	118° 4'12.72"W	200
36	11S	0400708	3734131	33°44'33.31"N	118° 4'19.02"W	200
37	11S	0400033	3732969	33°43'55.36"N	118° 4'44.77"W	159
38	11S	0399991	3733094	33°43'59.40"N	118° 4'46.46"W	379
39	11S	0400031	3733325	33°44'6.92"N	118° 4'45.00"W	171
40	11S	0400083	3733438	33°44'10.60"N	118° 4'43.02"W	367
41	11S	0400177	3733490	33°44'12.32"N	118° 4'39.39"W	328
42	11S	0400232	3733720	33°44'15.94"N	118° 4'37.30"W	151
43	11S	0400304	3733720	33°44'19.83"N	118° 4'34.55"W	300
44	11S	0400522	3733047	33°43'58.06"N	118° 4'25.81"W	336
45	11S	0400460	3733037	33°43'57.71"N	118° 4'28.21"W	300
46	11S	0400375	3733078	33°43'57.71"N	118° 4'31.51"W	238
47	11S	0400431	3732982	33°43'55.92"N	118° 4'29.32"W	337
48	11S	0400502	3732908	33°43'53.54"N	118° 4'26.53"W	396
49	11S	0400402	3732817	33°43'50.55"N	118° 4'30.38"W	377
50	11S	0400532	3732825	33°43'50.85"N	118° 4'25.33"W	300

UTM: (WGS84)

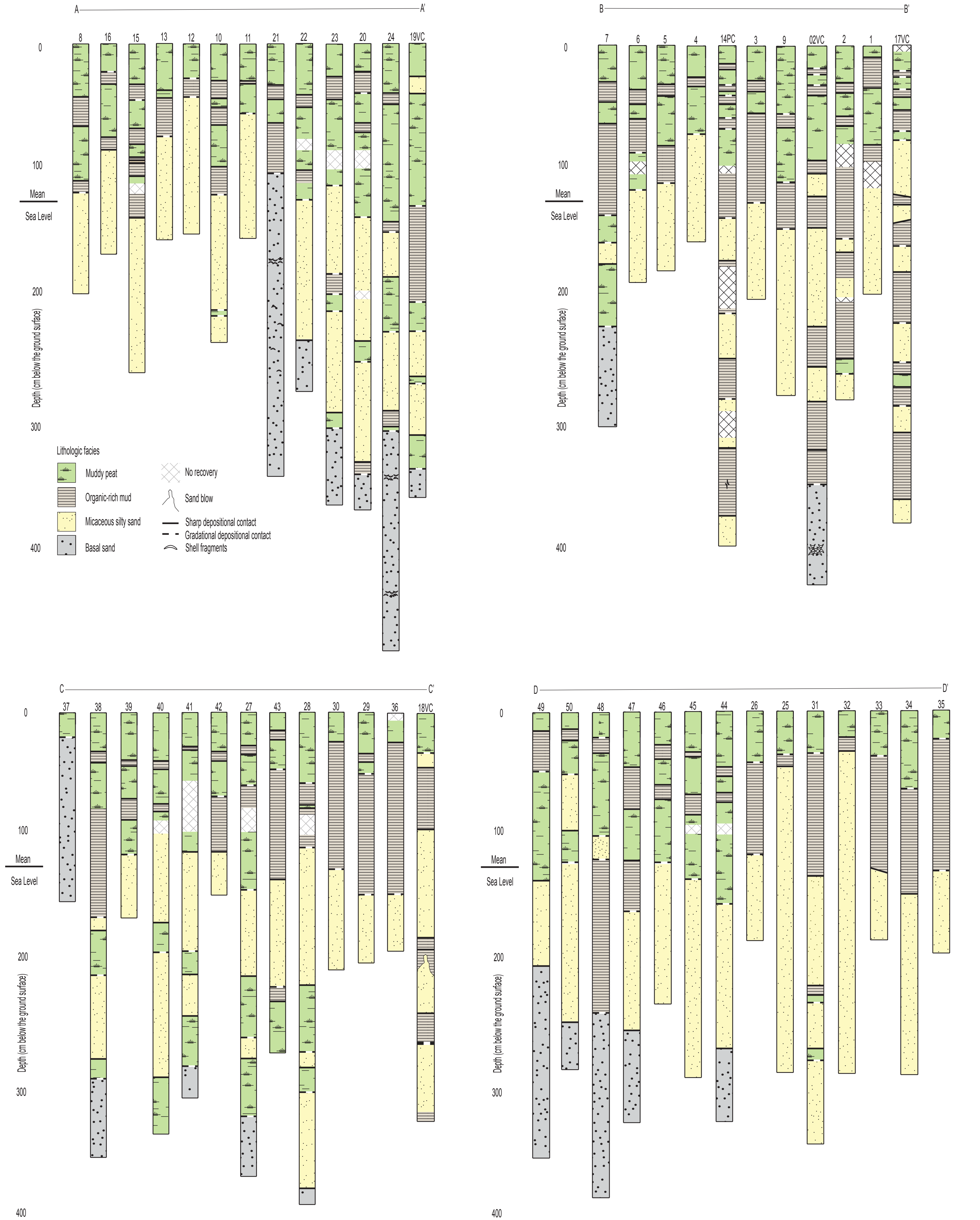

Supplementary Figure S3. Seal Beach saltmarsh lithostratigraphy across arbitrary transect lines shown on Figure 2b. The figure shows interlayered muddy peat and organic-rich mud units buried by coarse-grained micaceous silty sand. Reconnaissance cores 14, 19 (replaced by large diameter core lithostratigraphy), and piston cores 10 and 15 are not shown (cores not analyzed).

United States Department of the Interior

U.S. GEOLOGICAL SURVEY

Volcano Hazards Team
Global Climate Change Section
345 Middlefield Road
Menlo Park, CA 94025
25 April 2013

TO: Matthew Kirby, Department of Geological Sciences, CSU-Fullerton

FROM: Scott Starratt

SUBJECT: Preliminary analysis of samples from Seal Beach SB-02

Thirteen sediment samples from Seal Beach core SB-02 were analyzed for siliceous microfossils. Smear slides were made using Naphrax (r = 1.71).

Ten transects were scanned on each slide. All slides contained poorly to moderately well preserved microfossils. Most diatoms show evidence of physical breakage. This preservation is consistent with the preservation in marsh deposits around San Francisco Bay (SFB).

The number of specimens enumerated is insufficient for a statistical interpretation but does give you some idea of the environment of deposition.

16-17 cm

Caloneis westii – brackish (2-30 ppt); common in mudflats and salt marshes in SFB
Diploneis bombus – brackish (6-30+ ppt); rare in SFB; more common at Seal Beach
Diploneis smithii – slightly brackish to brackish (2-30+ ppt); found in SFB and saline lakes
Diploneis sp.
Gyrosigma eximium – brackish and fresh; found in mudflats and marshes in SFB
Gyrosigma sp.
Navicula sp.
Nitzschia granulata – marine to brackish; common in SFB
Surirella fastuosa – marine to brackish
Trachyneis aspera – brackish (15-30+ ppt)

19.5-20.5 cm

Eunotia sp. – freshwater, usually in acidic environments

24-25 cm

Caloneis westii
Diploneis bombus
Diploneis cf. *interrupta* – brackish (0-30+ ppt)

Diploneis oblongella – fresh to slightly brackish (0-10 ppt)
Diploneis smithii
Nitzschia granulata
Paralia sulcata – (5-30+ ppt); common in SFB
Surirella fastuosa
Trachyneis aspera

34-35 cm
Barren

37.5-38.5 cm
Aulacoseira sp. - freshwater
Nitzschia granulata

40-41 cm
Diploneis bombus
Diploneis cf. *interrupta*
Nitzschia granulata
Paralia sulcata
Trachyneis aspera
Sponge spicule

42-43 cm
Diploneis bombus
Nitzschia granulata
Paralia sulcata

45-46 cm
Diploneis cf. *interrupta*
Diploneis sp.
Nitzschia granulata
Trachyneis aspera

48-49 cm
Nitzschia granulata
Nitzschia sp.
Rhopalodia sp. – Fresh and brackish

52-53 cm
Caloneis westii
Diploneis bombus
Diploneis cf. *interrupta*
Diploneis smithii
Nitzschia granulata
Paralia sulcata
Surirella fastuosa

Trachyneis aspera

53.5-54.5 cm

Diploneis bombus
Nitzschia granulata
Paralia sulcata
Surirella sp.

57-58 cm

Diploneis bombus
Nitzschia granulata

74-75 cm

Barren

Supplementary Table S6. Radiocarbon data.

Number	Core ID	Depth (cm)	Material dated	[1]UCIAMS# / [2]CAMS#	^{14}C Age (BP)	2-Sigma	Removed from model
1	19VC	28.5	plant material	[1]168307	240 +/- 20	311-152	
2	02VC	39.5	plant material	[1]148130	375 +/- 20	500-320	
3	02VC	49.5	charcoal	[1]148131	1260 +/- 60	1281-1090	x
4	02VC	59.5a	plant material	[1]148132	615 +/- 25	654-554	
5	02VC	69.5	plant material	[1]148133	960 +/- 60	931-745	
6	02VC	79.5	plant material	[1]148134	1000 +/- 30	948-796	
7	02VC	99.5	plant material	[1]148135	850 +/- 30	897-690	
8	02VC	104.5	plant material	[1]148136	2595 +/- 50	2776-2545	x
9	02VC	111.5	plant material	[1]148137	2350 +/- 50	2459-2328	x
10	02VC	125.5	plant material	[1]145780	1395 +/- 35	1360-1270	
11	02VC	139.5	plant material	[1]148138	985 +/- 30	960-796	
12	02VC	190	charcoal	[1]134843	2295 +/- 20	2347-2326	x
13	02VC	220.5	plant material	[1]148139	2480 +/- 170	2361-2736	x
14	02VC	230.5	charcoal	[1]145781	1695 +/- 25	1694-1546	
15	02VC	248.5	plant material	[1]145782	1795 +/- 20	1736-1620	
16	02VC	256.5	plant material	[2]165539	1835 +/- 35	1865-1707	
17	02VC	268.5	charcoal	[1]128881	1775 +/- 20	1812-1642	
18	02VC	272	charcoal	[2]165540	1825 +/- 35	1864-1699	
19	02VC	274.5	charcoal	[2]165541	1955 +/- 30	1973-1826	
20	02VC	282.5	plant material	[2]165542	1990 +/- 45	2001-1827	
21	02VC	325.5	plant material	[1]148140	1045 +/- 25	964-932	x
22	02VC	375.5	plant material	[1]145783	3025 +/- 25	3255-3076	
23	02VC	394.5	shell	[1]128899	3755 +/- 15	3685-3431	
24	02VC	396.5a	shell	[1]128900	3865 +/- 15	3818-3555	
25	02VC	396.5b	shell	[1]128901	3435 +/- 15	3334-3115	
26	02VC	404.5	shell	[1]128902	3580 +/- 15	3466-3231	
27	18VC	171.5	plant material	[1]148141	2790 +/- 20	2925-2855	x
28	18VC	200.5	plant material	[1]148142	410 +/- 20	480-506	x
29	18VC	257.5	plant material	[1]148143	2150 +/- 20	2301-2061	
30	18VC	262.5a	plant material	[1]148144	2080 +/- 20	2119-1999	
31	18VC	262.5b	charcoal	[1]148145	2190 +/- 60	2339-2054	
32	18VC	288.5	plant material	[1]148146	3170 +/- 110	3300-2984	
33	14PC	210	plant material	[1]128882	1745 +/- 20	1709-1574	
34	14PC	277.5	plant material	[1]128883	2140 +/- 35	2304-2007	
35	14PC	333.5	plant material	[1]128884	2490 +/- 15	2717-2490	

[1]University of California, Irvine Accelerator Mass Spectrometry Laboratory ID Number

[2]Lawrence Livermore National Laboratory Center for Accelerated Mass Spectrometry Laboratory ID Number

Supplementary Data S7. Command lines for radiocarbon age model produced in OxCal v2.4[58].

```
Plot("Seal Beach Saltmarsh")
{
 Sequence("Stratigraphic Units")
 {
  Boundary("Base");
  Phase("Marine Embayment")
  {
   Curve("Marine13","Marine13.14c");
   Delta_R("LocalMarine",250,73);
   R_Date("02VC_404.5", 3580, 15);
   R_Date("02VC_396.5a", 3865, 15);
   R_Date("02VC_396.5b", 3435, 15);
   R_Date("02VC_394.5", 3755, 15);
  };
  Phase("Transitional Environment")
  {
   R_Date("02VC_375.5", 3025, 25);
   R_Date("18VC_288.5", 3170, 110);
   //R_Date("02VC_325.5",1045, 25);
  };
  Phase("Saltmarsh_1")
  {
   R_Date("14PC_333.5", 2490, 15);
   R_Date("14PC_277.5", 2140, 35);
   R_Date("18VC_262.5a", 2080, 20);
   R_Date("18VC_262.5b", 2190, 60);
   R_Date("18VC_257.5", 2150, 20);
 ar};
  Date("E3");
  Phase("Allochthonous Sediment")
  {
  R_Date("02VC_282.5", 1990, 45);
  R_Date("02VC_274.5", 1955, 30);
  R_Date("02VC_272", 1825, 35);
  R_Date("02VC_268.5", 1775, 20);
  R_Date("02VC_256.5", 1835,35);
  //R_Date("02VC_220.5", 2480, 170);
  };
  Phase("Saltmarsh_2")
  {
   R_Date("02VC_248.5", 1795, 20);
   R_Date("02VC_230.5", 1695, 25);
   R_Date("14PC_210", 1745, 20);
   //R_Date("18VC_200.5", 410, 20);
  };
  Date("E2");
  Phase("Allochthonous Sediment")
```

```
  {
   //R_Date("02VC_190", 2295, 20);
   //R_Date("02VC_111.5", 2350, 50);
   //R_Date("02VC_104.5", 2595, 50);
   //R_Date("18VC_171.5", 2790, 20);
   R_Date("02VC_125.5", 1395, 35);
   R_Date("02VC_139.5", 985, 30);
  };
  Phase("Saltmarsh_3")
  {
   R_Date("02VC_99.5", 850, 30);
   R_Date("02VC_79.5", 1000, 30);
   R_Date("02VC_69.5", 960, 60);
   R_Date("02VC_59.5", 615, 25);
   //R_Date("02VC_49.5", 1260, 25);
  };
  Date("E1");
  Phase("Saltmarsh_4")
  {
   R_Date("02VC_39.5", 375, 20);
   R_Date("19VC_28.5", 240, 20);
  };
  Boundary("Top");
 };
};
```
